# Comparative analysis of tissue-specific genes in maize based on machine learning models: CNN performs technically best, LightGBM performs biologically soundest

**DOI:** 10.3389/fgene.2023.1190887

**Published:** 2023-05-09

**Authors:** Zijie Wang, Yuzhi Zhu, Zhule Liu, Hongfu Li, Xinqiang Tang, Yi Jiang

**Affiliations:** ^1^ School of Agriculture, Sun Yat-sen University, Shenzhen, China; ^2^ School of Intelligent Systems Engineering, Sun Yat-sen University, Shenzhen, China

**Keywords:** tissue specific genes, maize, CNN, limma, SHAP (shapley additive explanation), Lightgbm

## Abstract

**Introduction:** With the advancement of RNA-seq technology and machine learning, training large-scale RNA-seq data from databases with machine learning models can generally identify genes with important regulatory roles that were previously missed by standard linear analytic methodologies. Finding tissue-specific genes could improve our comprehension of the relationship between tissues and genes. However, few machine learning models for transcriptome data have been deployed and compared to identify tissue-specific genes, particularly for plants.

**Methods:** In this study, an expression matrix was processed with linear models (Limma), machine learning models (LightGBM), and deep learning models (CNN) with information gain and the SHAP strategy based on 1,548 maize multi-tissue RNA-seq data obtained from a public database to identify tissue-specific genes. In terms of validation, V-measure values were computed based on k-means clustering of the gene sets to evaluate their technical complementarity. Furthermore, GO analysis and literature retrieval were used to validate the functions and research status of these genes.

**Results:** Based on clustering validation, the convolutional neural network outperformed others with higher V-measure values as 0.647, indicating that its gene set could cover as many specific properties of various tissues as possible, whereas LightGBM discovered key transcription factors. The combination of three gene sets produced 78 core tissue-specific genes that had previously been shown in the literature to be biologically significant.

**Discussion:** Different tissue-specific gene sets were identified due to the distinct interpretation strategy for machine learning models and researchers may use multiple methodologies and strategies for tissue-specific gene sets based on their goals, types of data, and computational resources. This study provided comparative insight for large-scale data mining of transcriptome datasets, shedding light on resolving high dimensions and bias difficulties in bioinformatics data processing.

## 1 Introduction

Tissue-specific genes are a class of genes whose expression and activity are preferential in one or more tissues or cell types (Xiao et al., 2010). The identification of these genes advances our understanding of the relationship between tissues and genes, as well as the discovery of novel tissue-specific molecular targets. One of the methods for identifying tissue-specific genes is to apply a linear statistical model, such as Limma (Ritchie et al., 2015) and edgeR (Robinson et al., 2010), to discover differentially expressed genes (DEGs) between pairs of tissues through transcriptome data and subsequently validate their tissue specificity. In addition to the models described above that are based on empirical Bayesian estimation, researchers have created novel variable linear algorithms for expression data. For example, Vasiliu et al. used penalized Euclidean distance (PED) to analyze data from RNA-seq and other global expression experiments with small sample sizes and high dimensionality (Vasiliu et al., 2015).

Meanwhile, RNA-seq (RNA sequencing) has allowed researchers to validate gene expression across the entire genome and develop a system-level understanding of biological processes. More and more RNA-seq data has been generated, and multiple comprehensive databases of transcriptome data have been created, providing researchers with an abundance of resources for finding key tissue-specific genes through gene expression. However, identifying relevant genes in high dimensionality and variance transcriptome data remains difficult, limiting the utility of these tens of thousands of publicly available gene expression datasets (Kong et al., 2011). Nevertheless, with the application of artificial intelligence (AI), machine learning models, together with special explanation methods for model interpretation, have proven remarkable accuracy and efficiency in training with the full datasets via a transcriptome database. Based on their different algorithms and training strategies, machine learning models show distinct performance and capability, for example, Light Gradient Boosting Machine (LightGBM) (Ke et al., 2017) is a framework for machine learning that uses gradient boosting and decision trees, which aims to be efficient and scalable by using techniques like Gradient-based One Side Sampling and Exclusive Feature Bundling, and Convolutional Neural Network (CNN) is a deep learning neural network designed for processing structured arrays of data (Yap et al., 2021), containing many convolutional layers that are capable of recognizing more sophisticated shapes (Deng et al., 2021; Liu and Zhang, 2022). In this case, researchers used numerous machine learning models to mine expression datasets beyond traditional methods. Sun Kim et al. developed an ensemble model that included network information such as network propagation and network property to identify DEGs, which rated top in detecting ground truth (GT) genes in eight datasets obtained from the GEO database (Moon et al., 2022). Furthermore, Maciej and Nicola et al. developed a convolutional neural network to predict tissue classification using Genotype-Tissue Expression (GTEx) RNA-seq data from 47 tissues. The classifier attained an average F1 score of 96.1% on holdout GTEx data, and the 2,423 most discriminating genes were identified using SHAP values (Yap et al., 2021). In particular, the developing methodologies of machine learning and neural networks outperformed classic statistical models and were able to manage more extensive and complicated database data.

However, in comparison to human and mouse research, plant transcriptome data are rather limited. For a variety of reasons, benchmarking methods have yet to be used to identify tissue-specific genes in plants. First, there is still noise and batch effects in transcriptome datasets. In contrast to the common use of single-cell RNA-seq for human research, the predominant strategy for plants is still bulk RNA-seq, which involves multi-cell sequencing and contains a lot of noise and technical variation. Second, the tissue type distribution in RNA-seq data is unbalanced. Because of the accessibility and choice for plant tissues in research, the majority of RNA-seq data is derived from leaves and roots, and many experiments have focused on mixing tissue sequencing. Because of the imbalanced tissue types, downstream analyses would be biased. Third, due to the scarcity of transcriptome data for each species, techniques and models are limited to identifying tissue-specific genes. For the complexity and diversity of plant genomes, tissue-specific genes discovered in restricted studies are not convincing and universal. As a result, despite the establishment of many plant transcriptome datasets in recent years, it remains difficult to use tissue-specific gene identification methodologies, particularly machine learning models, due to biases, overfitting, and imbalance (Ma et al., 2014b; Dorneanu et al., 2022). As a technical gap, unique methodologies for identifying tissue-specific genes utilizing the whole plant transcriptome database for particular species have never been implemented, necessitating further comparison and validation.

Because of the importance of maize in modern crop breeding and its comparatively extensive transcriptome data, the widely produced crop maize (*Zea mays*) was chosen as the target plant in this study for methodologies comparison on tissue-specific gene identification (Chen et al., 2020). Implementing a comprehensive and comparative study on maize transcriptome data was significant because it would serve as the first example of evaluating different tissue-specific gene identification methods on plants, contributing to a better understanding of maize molecular and functional differences between tissues.

**FIGURE 1:**
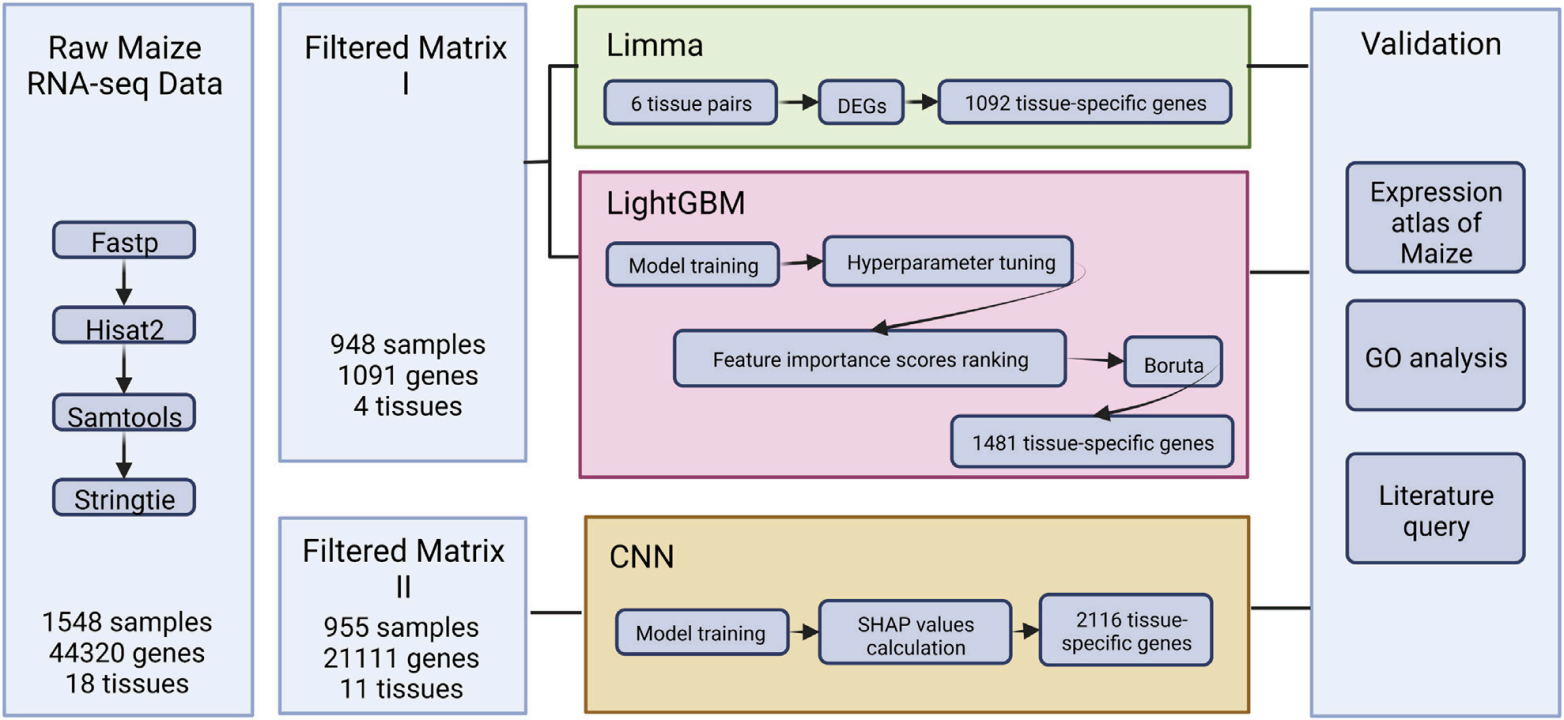
The overview of the study.

In this study, we aimed to evaluate the performance of the linear model [Limma (Ritchie et al., 2015)], machine learning model [LightGBM (Ke et al., 2017)], and the deep learning model [CNN (Yap et al., 2021)] in identifying tissue-specific genes from maize transcriptome data. Initially, the importance of identifying tissue-specific genes and mining transcriptome data with machine learning algorithms was emphasized. Furthermore, the existing technique gaps and issues in analyzing plant transcriptome datasets were investigated (Section 1. *Introduction*). The following section presented the training data acquisition, three model training processes, and biological and statistical evaluation methodologies. (Section 2. *Material and methods*; Figure 1). Furthermore, the performance of the three techniques was compared, and the core set of tissue-specific genes in maize was integrated further to uncover key loci for maize growth and differentiation (Section 3. *Results*). Section 4 analyzed the efficacy of further benchmarking machine learning models and conducted a thorough discussion of the elements that contributed to the various tissue-specific gene sets created by each technique.

## 2 Materials and methods

### 2.1 Maize RNA-seq collection and gene expression calculation

Considering the insufficient RNA-seq collection and inconvenient processing pipeline for maize expression through multiple distinctive experiments, a comprehensive set of maize RNA-seq data were obtained from public databases, using the search terms “Maize RNA-seq” on NCBI (Barrett et al., 2013) and ENA (Harrison et al., 2021). Then, standard RNA-seq files processing pipeline (Pertea et al., 2016) written in Python (3.9), comprised of packages Fastp (0.23.2) (Chen et al., 2018), Hisat2 (2.2.1) (Kim et al., 2019), Samtools (1.16.1) (Li et al., 2009), and Stringtie (2.2.1) (Pertea et al., 2015), was implemented for quality control, alignment, and transcripts per million (TPM) value calculation across all samples.

In addition, the imbalanced tissue types and redundant genes were removed. MCScanX (Wang et al., 2012) was used to filter out the maize genes that were colinear with sorghum for later training.

### 2.2 Limma for DEGs identification among tissue pairs

DEG analysis provided the key to discovering tissue-specific genes using the linear model. First, differentially expressed genes would be identified by comparing each tissue pair initially. Then the integration of these genes would be the tissue-specific gene set. In this stage, the R:Limma (3.54.1) (Ritchie et al., 2015), which was accessible for TPM values processing, was utilized to control the variable in later comparison, as opposed to typical methods like edgeR and DESeq, which require a counts table as input.

**FIGURE 2:**
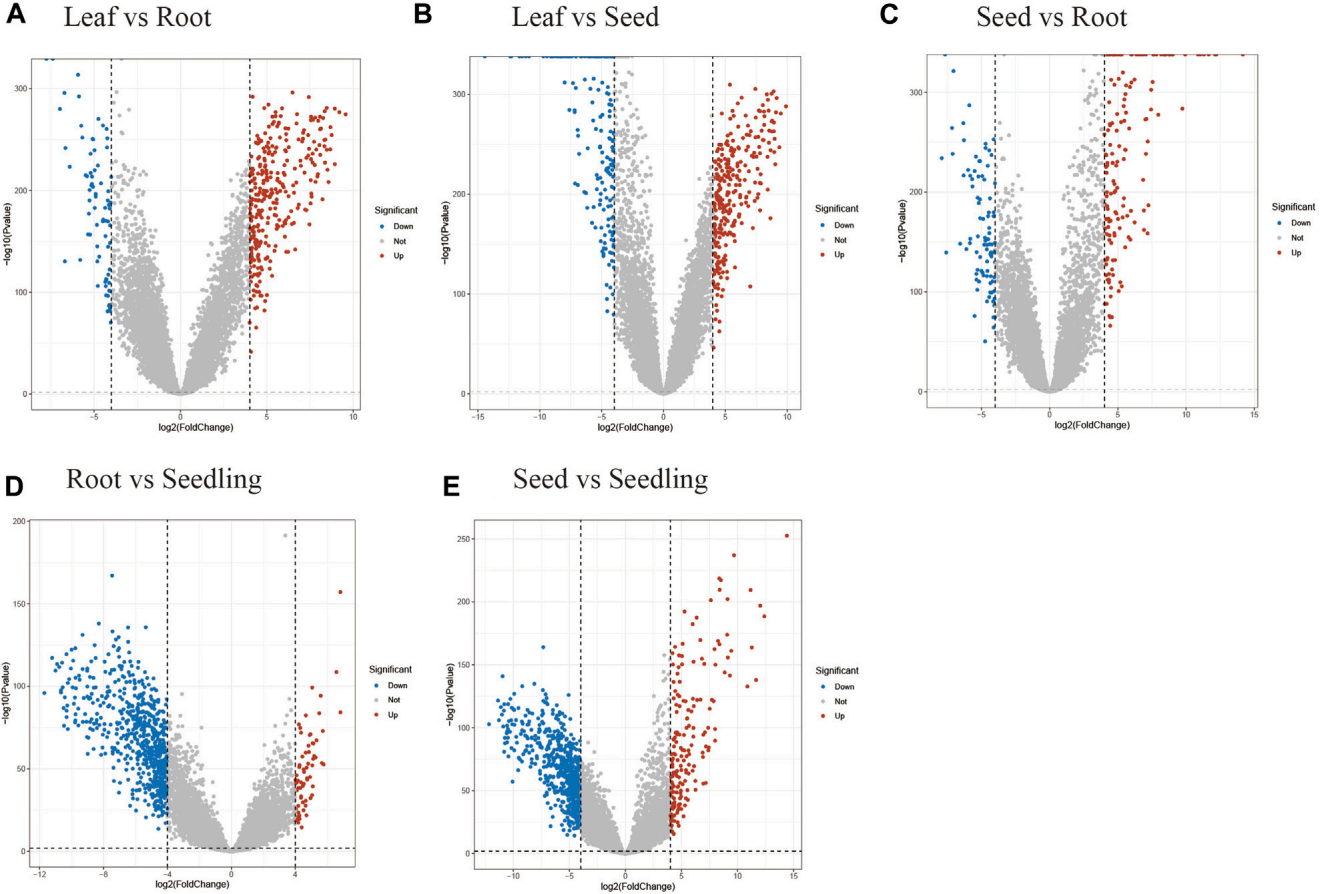
Volcano plots for the DEGs between tissue pairs. The plot for the “Leaf-Seedling” pair wasn’t shown for only 2 DEGs identified in this situation. **(A)** The differentially up-regulated and down-regulated genes between leaf and root. **(B)** The differentially up-regulated and down-regulated genes between leaf and seed. **(C)** The differentially up-regulated and down-regulated genes between seed and root. **(D)** The differentially up-regulated and down-regulated genes between seedling and root. **(E)** The differentially up-regulated and down-regulated genes between seed and seedling.

Through the process, DEGs were discovered between 6 tissue pairs, including “Leaf-Root”, “Leaf-Seed”, “Leaf-Seeding”, “Root-Seed” and “Seed-Seeding”. First, the log2 values of TPM were transformed. Then the indexes of Fold Change (logFC), Average Expression (AveExp), and Adjusted *p*-value (adj.P.Val) for each gene were calculated using the default parameters of Limma. Because the goal was to uncover tissue-specific genes, the DEGs filtering criterion was set to be strict, resulting in the selection of only genes with adj.P.Val less than 0.01 and an absolute value of logFC more than 4 (Figure 2). Furthermore, the positive and negative logFC values were used to categorize the upregulated and downregulated genes (Supplementary Table S3). Finally, the DEGs discovered in six tissue pairs were merged to reflect maize tissue-specific genes in relation to the Limma tissue-specific genes collection (Supplementary Table S4).

### 2.3 LightGBM machine learning method

In this stage, the Scikit-learn-based Pycaret (3.0) (Gain and Hotti, 2021) module was implemented in the Python 3.9 environment for LightGBM (Ke et al., 2017). First, the TPM values were normalized to obtain unbiased results, and the SMOTE method (Chawla et al., 2002) was utilized to adjust imbalanced samples, particularly for the seedling tissue type. Then the LightGBM ensemble learning model was used to train the input expression matrix, 70% of which was used as the training set and 30% as the validation set. In addition, number of folds to be used in cross validation was set as 10 by the “stratifiedkfold” strategy as default. The hyperparameters were further updated automatically using the grid search approach with the function “tune_model” in Pycaret to generate a robust performance and high accuracy training model.

The Booster module’s function “feature importance scores” was imported to extract the LightGBM training model’s features based on information gain (Silva et al., 2021) (Supplementary Table S5). Furthermore, the BORUTA method, which is based on information gain, was used to filter out noisy feature genes in order to estimate the credible threshold of feature scores in the gene list for further validation. To implement the BORUTA algorithm, each feature gene in the original matrix was shuffled. The shuffled shadow characteristics were combined with the original real features to create a new training matrix. The new training matrix was then used as input to train a decision tree model, and feature importance scores were generated as well. In addition, the Z-scores were calculated for each real feature and shadow feature respectively, according to the equation,

$$\left\{\begin{array}{l} Z_{score,real}=\frac{{\left(\bar{feature\_impor\tan ce\_score}\right)}_{real}}{\sigma_{feature\_impor\tan ce\_score,real}}\\ Z_{score,shadow}=\frac{{\left(\bar{feature\_impor\tan ce\_score}\right)}_{shadow}}{\sigma_{feature\_impor\tan ce\_score,shadow}}\\ Z_{\max}=\max\left(Z_{score,shadow}\right) \end{array}\right.$$ (1)

where Z_max_ was defined as the largest value among the shadow features. Real features with Z_score, real_ larger than Z_max_ would be kept while the smaller ones would be regarded as the noise features as random values and discarded. After filtering, the result was regarded an LGBM tissue-specific gene set (Supplementary Table S6).

The training and performance of other 13 machine learning models (Logistic Regression, K Neighbors Classifier, Naive Bayes, Decision Tree Classifier, SVM-Linear Kernel, Ridge Classifier, Random Forest Classifier, Quadratic Discriminant Analysis, Ada Boost Classifier, Gradient Boosting Classifier, Linear Discrimination Analysis, Extra Trees Classifier and Dummy Classifier) were implemented by the “compare_models” function in Pycaret with the same parameters.

### 2.4 CNN architecture and training

To address the issue of large dimensionality and avoid overfitting, the training matrix was altered to fit the convolutional neural network model (CNN) architecture while the data dimension was extensively recreated. Because CNN performance was equivalent when using imbalanced and balanced training data (Yap et al., 2021), all tissues with more than ten samples in the original expression matrix were retained, and non-colinear and low expression genes were still removed from the new matrix. Then the genes left could be zero-padded into a square vector that appeared as pixelated images to accommodate the CNN design.

**FIGURE 3:**
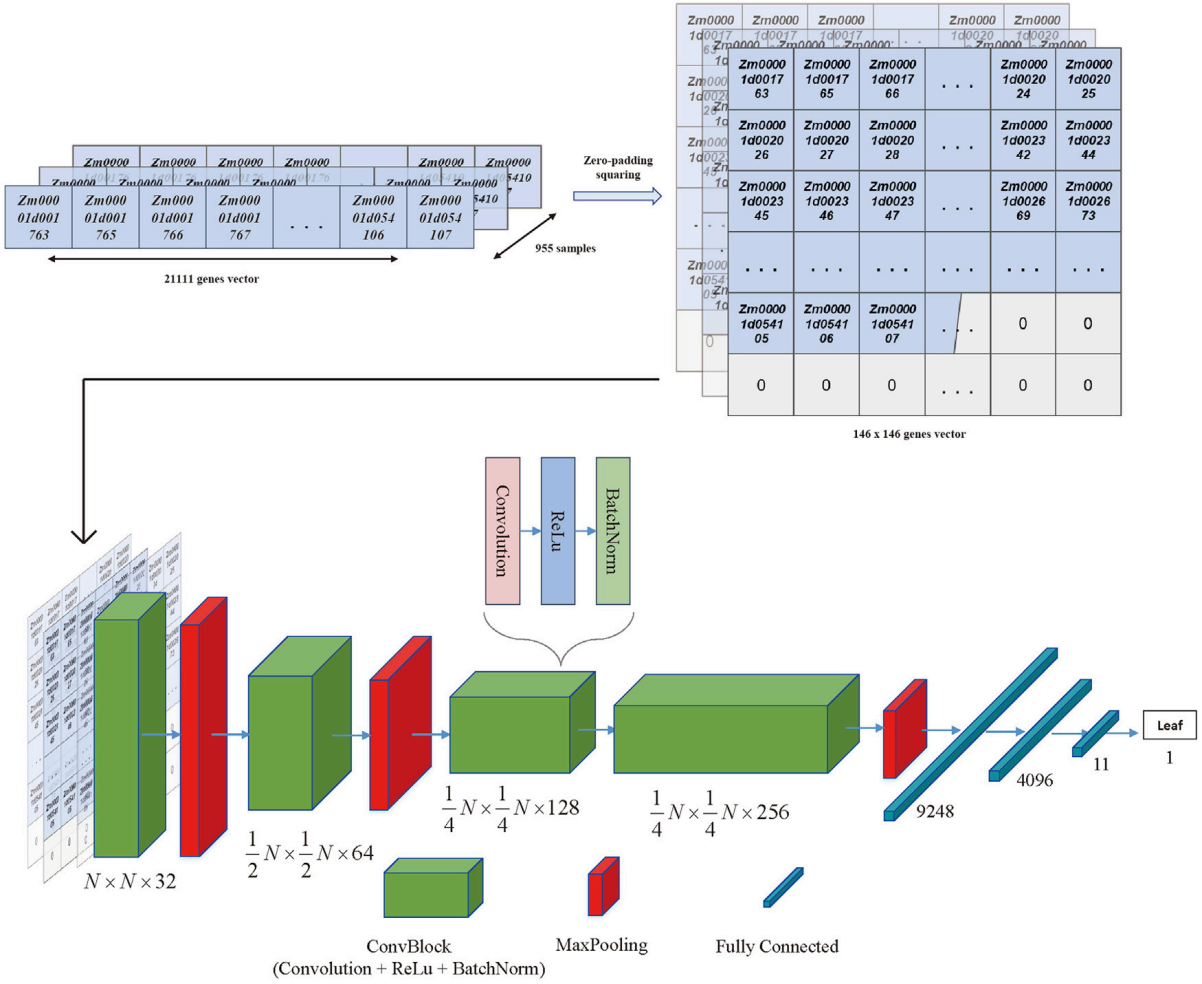
The CNN model architecture. The 21,111 genes vector for each sample was transferred to the 146*146 vector with zero padding and squaring. Then the 955 squared vectors were seen as the input data of the CNN architecture. The architecture of the CNN included the convolutional blocks, max pooling blocks, and fully connected blocks, which were organized as the figure shown. The output of the architecture was the tissue type that obtained the highest probability in the last layer.

Moreover, the CNN training model was built by TensorFlow (2.11) in the manner of the previous literature (Yap et al., 2021), as shown in Figure 3, where a ten-layer CNN model was carefully created for robust and accurate training. Each convolution block (ConvBlock) in the architecture was a layer stack that included a convolution layer with kernel shaped in (3,3), activation layer (Rectified Linear Unit [ReLU]), and normalization layer (Batch Normalization [BatchNorm]). Furthermore, MaxPooling was a downsampling layer, whereas the fully connected (dense) layer flattened the preceding matrix into a single vector. Finally, the hidden layer network produced one of 11 classes known as the most likely tissue type. For the multi-classification task, the categorical cross entropy was chosen as the loss function, and the formula was as follows:

$$loss=-\sum_{i=1}^{N}y_{i}\cdot \log{\hat{y}}_{i}$$ (2)

where y_i_ was the ground truth label with 1 as right prediction and 0 as wrong prediction (Yu et al., 2020). The proportion of the training matrix and validation matrix was set as 8:2. During the training, the root mean square prop algorithm (RMSprop) was used as the optimizer, while setting the learning rate as 0.0001, the rho value as 0.9 and the decay as 0.01 for the efficiency and the accuracy of the results.

To get a constant accuracy score and loss value for the validation set, the model’s hyperparameters were slightly adjusted due to the total accuracy values and loss. Finally, the model was trained with 64 batches and 50 epochs.

### 2.5 SHAP values calculation

To identify the feature genes driving CNN’s learning processes, the GradientExplainer function from the SHapley Additive exPlanations (SHAP) package (0.41.0) was employed.

The weight and architecture of the CNN model were loaded into the SHAP explainer to initialize it with the training expression matrix. Following initialization, the test data set was fed into the SHAP explainer, yielding an array of SHAP values for each sample. Only accurately predicted samples were preserved in the SHAP array, and only the associated SHAP value of the correct class was chosen, resulting in a single value per gene per sample. To evaluate global relevance, the median SHAP value for each gene within each tissue was calculated and ranked from highest to lowest. The 1% most highly ranked genes per tissue were put into the CNN tissue-specific gene set (Supplementary Tables S7, S8).

### 2.6 Comparison of the tissue-specific gene sets based on clustering and V-measure

Utilizing the Limma gene set, LGBM gene set, SHAP gene set, complete gene set, and randomly selected gene set as clustering features, all samples were grouped into 11 groups using the k-means method (Hartigan and Wong, 1979). The groups were then visualized with the Uniform Manifold Approximation and Projection (UMAP) dimension reduction technique (McInnes and Healy, 2018) (Supplementary Figure S3) and quantitatively evaluated with V-measure (Rosenberg and Hirschberg, 2007).

The V-measure analysis was carried out in two steps to statistically compare the three tissue-specific gene identification approaches. To begin, V-measure values for k-means clustering were computed. The distribution was illustrated for the Limma, LGBM, and SHAP gene sets, as well as five randomly selected gene sets. The harmonic mean of homogeneity (h) and completeness (c) for the categories division is the V-measure value (v) (Rosenberg and Hirschberg, 2007), which may be determined as follows:

$$\begin{array}{c} \left\{\begin{array}{l} H\left(C|K\right)=-\sum_{k=1}^{\left|K\right|}\sum_{c=1}^{\left|C\right|}\frac{n_{c,k}}{n}\log\left(\frac{n_{c,k}}{n_{k}}\right)\\ H\left(C\right)=-\sum_{c=1}^{\left|C\right|}\frac{n_{c}}{n}\log\left(\frac{n_{c}}{n}\right)\\ H\left(K|C\right)=-\sum_{c=1}^{\left|C\right|}\sum_{k=1}^{\left|K\right|}\frac{n_{c,k}}{n}\log\left(\frac{n_{c,k}}{n_{k}}\right)\\ H\left(K\right)=-\sum_{k=1}^{\left|K\right|}\frac{n_{c}}{n}\log\left(\frac{n_{c}}{n}\right)\\ h=1-\frac{H\left(C|K\right)}{H\left(C\right)}\\ c=1-\frac{H\left(K|C\right)}{H\left(K\right)}\\ v=\frac{2\times h\times c}{h+c} \end{array}\right. \end{array}$$ (3)

In the above formula, n represents the total number of samples, n_c_ represents the number of samples in a specific correct category, n_k_ represents the number of samples in the corresponding predicting category and n_c,k_ represents the number of samples in the c category which are divided to the k predicting category correctly. The greater the V-measure value, the better the performance of the feature set, which identifies more traits and distributions between categories.

Furthermore, it was important to assess the likelihood of randomly selecting gene sets as well as tissue-specific gene identification procedures, which would demonstrate the resilience and soundness of specific techniques in another aspect. As a result, 100 random gene samplings were carried out using various random k-means initializations. The mean of each k-means sample distribution was used to create a null distribution. The “true” test statistic would be the mean values from the k-means sampling of tissue-specific genes identification technique. The probability of picking SHAP genes at random was then evaluated using a one tail Student’s t-test.

### 2.7 Maize genes expression validation

The maize development atlas (Walley et al., 2016), which was excluded from the training set, was used as a validation set because it was searchable and the expression could be read with ease using MaizeGDB (Portwood et al., 2019). Moreover, the chosen genes’ molecular activities were queried in UniProt (Dogan, 2019), and relevant literature was acquired to demonstrate the gene’s roles and significance.

### 2.8 GO analysis

Gene Ontology (GO) enrichment analysis was conducted via the ShinyGO (v0.76) platform (Ge et al., 2019), with additional data visualization procedures enabled by R package clusterProfiler (v4.6.0) (Wimalanathan et al., 2018; Wu et al., 2021). With respect to the GO dataset, both experimental (EXP) and phylogenetically inferred (IBA) evidence codes were utilized. Nevertheless, only biological process (BP) datasets were used concerning the scope of the study.

### 2.9 Computational resources

The raw RNA-seq data processing and expression matrixes generation were applied on the local Linux server with 64 cores Intel(R) Xeon(R) Gold 5218 CPU @ 2.30 GHz. The machine learning models and CNN tasks were carried out on the online server with a 12 GB TITAN Xp GPU.

## 3 Results

### 3.1 Overview of the maize training matrix

From the public databases, 1,548 fastq format files across 18 maize tissues were obtained (Supplementary Table S1) and an expression matrix with 1,548 rows (samples) and 44,320 columns (genes) was constructed totally. Among the processes, the average count value of all RNA-seq was 10^8^ with an average Q20 of 95% (Supplementary Figure S1A). And Hisat2 aligned all sequences to the B73 v4 reference genome with an average 91% alignment rate (Supplementary Figure S1B). These consequences showed that the maize multi-tissue expression matrix was of good quality and standardization.

The original tissue type labels from public databases were imbalanced. However, the machine learning model LightGBM required balanced samples and an appropriate train-test set splitting. Therefore, the tissues with less than 10 sample recordings and the ‘mixed’ tissue type were discarded, while the remaining samples were categorized as “Leaf,” “Seed,” “Root” and “Seeding” according to their organs. Besides tissue types, there were also low-expression and biologically insignificant genes existing among the original genes in the matrix. According to former literature, only the genes colinear with sorghum were chosen in this study, for they would reflect more significant biological functions and reduce the dimensions of later machine learning model training. After selected by MCScanX (Wang et al., 2012), only the filtered genes with TPM larger than one were left, making the training matrix with 948 rows and 21,091 columns consequently (Supplementary Table S2).

### 3.2 Tissue-specific genes set identification by Limma

Limma discovered the differentially expressed genes in maize tissue pairs in order to create a collection of tissue-specific gene sets. Except for the “Leaf-Seeding” pair, which had only two differentially expressed genes, all tissue pairs had approximately 500 genes each (Table 1). Because the filtering threshold was set relatively strictly, the DEGs discovered might be considered tissue-specific genes. As a result, by taking the intersection of the six DEGs groups, 1,092 genes were gathered as the Limma tissue-specific gene set. The smaller number of DEGs in the “Leaf-Seedling” pair may be due to the high relevance of these two tissues and the limited sample size of seedlings.

**TABLE 1:** Numbers of DEGs among 6 tissue pairs.

Tissue pairs	Total DEGs	Upregulated DEGs	Downregulated DEGs
Leaf-Root	390	313	77
Leaf-Seed	462	281	182
Leaf-Seeding	2	0	2
Root-Seeding	710	68	643
Root-Seed	253	161	93
Seed-Seeding	799	181	619

**FIGURE 4:**
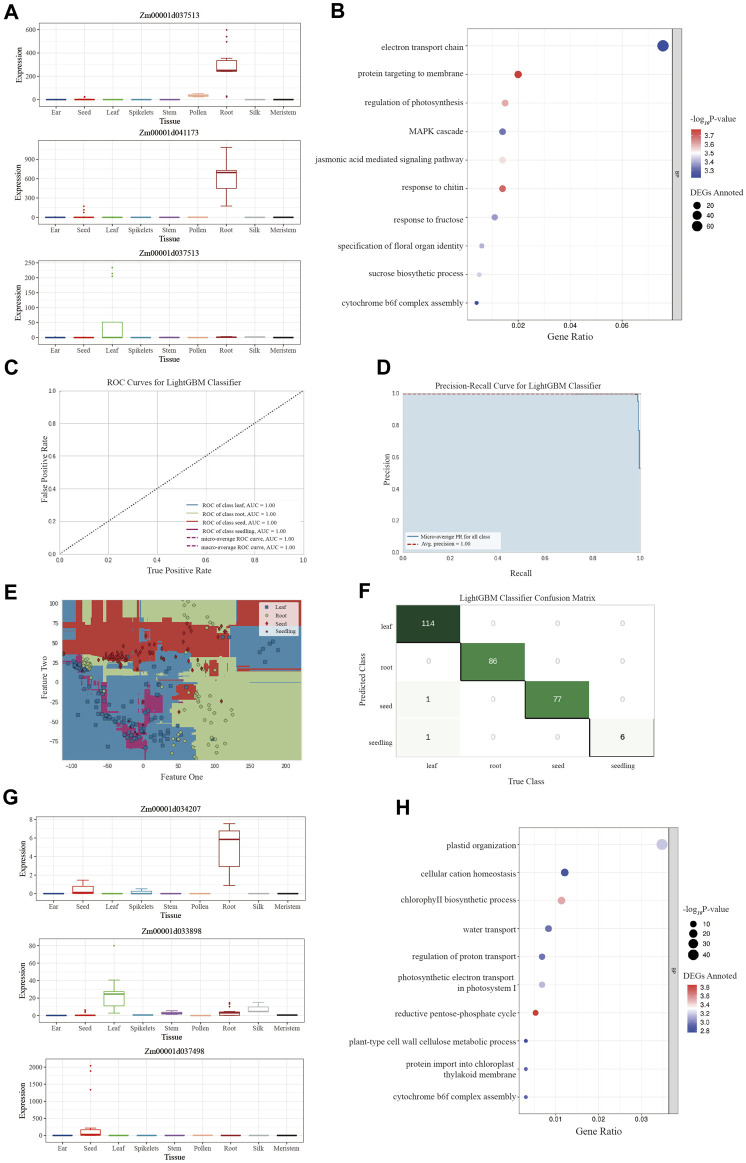
The evaluation plots of Limma and LightGBM models. **(A)** The expression bar plot of the top-rank Limma genes queried from the maize expression atlas in MaizeGDB. **(B)** The biological process of the Limma tissue-specific gene set clustered by the GO analysis. **(C)** The ROC curve of the LightGBM model after training on the maize multi-tissue expression data. **(D)** The PR curve of the LightGBM model. **(E)** The confusion matrix of the LightGBM model validation set. It could be seen that a few samples from seed and seedling were mismatched into the leaf set. **(F)** The boundary plot of the LightGBM model for classification. It could be seen that the relation between leaf and seedling samples was relatively close due to the mismatching of the model. **(G)** The expression bar plot of the top 3 rank LGBM genes queried from the maize expression atlas in MaizeGDB. **(H)** The biological process of the LGBM tissue-specific gene set clustered by the GO analysis..

MaizeGDB was then used to retrieve gene information. The majority of the genes discovered were particularly expressed in one kind of tissue; for example, the downregulated genes discovered in the ‘Leaf-Seeding’ pair were both specifically expressed in mature leaves and had previously been described in research articles (Figure 4A). Furthermore, the biological processes involved in the DEGs discovered in each tissue pair were mostly related to the functions of specific tissues (Supplementary Table S9). For example, when comparing leaf and root, Limma discovered DEGs involved in “NADPH regeneration,” “water transport,” and “PSII associated light-harvesting complex Il catabolic process,” in addition to some general functions of “translation” and “protein autophosphorylation” (Figure 4B).

### 3.3 Tissue-specific genes identification by LightGBM

Using maize expression data, 14 kinds of machine learning model were trained to perform tissue classification (Table 2). LightGBM got the first rank due to its remarkable accuracy rate near 0.99 and highest Area Under the Curve (AUC) value, indicating that it could handle the false positive prediction properly and evaluated samples reasonably in the case of unbalanced samples.

**TABLE 2:** The evaluation criteria for 14 machine learning models.

Model	Accuracy	AUC^a^	Recall	Prec^b^	F1^c^	Kappa^d^	TT^e^
Light Gradient Boosting Machine	0.9895	0.9998	0.9462	0.9877	0.9878	0.9842	68.718
Logistic Regression	0.991	0.9984	0.9474	0.9891	0.9893	0.9865	3.019
Random Forest Classifier	0.9895	0.9981	0.9349	0.9848	0.9866	0.9842	0.917
Extra Trees Classifier	0.991	0.9976	0.9474	0.9891	0.9893	0.9865	1.065
Gradient Boosting Classifier	0.9789	0.996	0.9268	0.9782	0.9775	0.9685	153.012
K Neighbors Classifier	0.9864	0.9959	0.967	0.9895	0.9871	0.9798	1.086
Naive Bayes	0.9759	0.9814	0.8429	0.967	0.9705	0.9638	0.887
Decision Tree Classifier	0.9698	0.9775	0.8718	0.965	0.9661	0.9547	1.33
Ada Boost Classifier	0.6779	0.966	0.6942	0.7457	0.661	0.5924	8.818
Linear Discriminant Analysis	0.6029	0.7268	0.603	0.6543	0.609	0.4357	1.593
Quadratic Discriminant Analysis	0.565	0.7017	0.4901	0.3913	0.4428	0.3945	1.17
Dummy Classifier	0.4003	0.5	0.25	0.1603	0.2289	0	0.766
SVM - Linear Kernel	0.991	0	0.9474	0.9891	0.9893	0.9865	0.878
Ridge Classifier	0.991	0	0.9474	0.9891	0.9893	0.9865	0.872

^a^ AUC: area under curve, the area under the ROC, curve.

^b^ Prec.: precision.

^c^ F1: F1-score, the harmonic mean of precision and recall value.

^d^ Kappa: Kappa-value, measuring model evaluation accuracy in multiple classifications.

^e^ TT: the processing time for the 10-fold training.

As a result, LightGBM was chosen to represent non-neural-network-based machine learning method to identify tissue-specific genes. After 10 rounds of cross-validation training, the mean values of accuracy, AUC, and F1 score were 0.9909, 0.9998, and 0.9917, respectively (Table 3). The model was shown to be surprisingly accurate, based on the Receiver Operating Characteristic (ROC) and Precision-Recall (PR) plots (Figures 4C, D), despite a few mismatches between seedling and leaf samples in the test set due to their close association (Figures 4E, F).

**TABLE 3:** The evaluation criteria for LightGBM in 10 rounds of cross-validation training.

	Accuracy	AUC^a^	Recall	Prec^b^	F1^c^	Kappa^d^
0	0.9851	0.9995	0.9844	0.9888	0.9859	0.9781
1	1	1	1	1	1	1
2	0.9697	0.9994	0.9719	0.9717	0.9698	0.9544
3	1	1	1	1	1	1
4	0.9848	1	0.9844	0.9854	0.9847	0.9772
5	1	1	1	1	1	1
6	0.9697	0.9986	0.8618	0.9711	0.9673	0.9544
7	1	1	1	1	1	1
8	1	1	1	1	1	1
9	1	1	1	1	1	1
Mean	0.9909	0.9998	0.9802	0.9917	0.9908	0.9864
SD	0.0121	0.0004	0.0406	0.0113	0.0125	0.0182

^a^ AUC: area under curve, the area under the ROC, curve.

^b^ Prec.: precision.

^c^ F1: F1-score, the harmonic mean of precision and recall value.

^d^ Kappa: Kappa-value, measuring model evaluation accuracy in multiple classifications.

^e^ TT: the processing time for the 10-fold training.

Further analysis was conducted for the major feature genes created by LightGBM, which were chosen to be the nodes of the classification model to distinguish different tissues, and 1,481 genes were finally identified as LGBM tissue-specific genes using the Boruta algorithm (Figure 4G). Their biological processes were clustered using GO analysis (Figure 4H; Supplementary Table S10). According to the findings, the majority of LGBM tissue-specific expression genes involved in pigment biosynthesis, photosynthetic electron transport, and chloroplast formation.

The high-ranking LGBM genes were queried in MaizeGDB to see if they were only expressed in one tissue, and supporting literature was collected to corroborate their significance (Supplementary Figure S2). As a result, as expected, the majority of LGBM genes were tissue-specifically expressed, and it was noteworthy that the majority of genes found in the top rank were transcription factor genes, which have previously been well studied, indicating that they may be essential elements in maize gene expression regulation (Table 4).

**TABLE 4:** The information of the top 10 genes in the LGBM tissue-specific gene set.

Gene ID	Gene	Specific expression	Related papers (top three)
Zm00001d037498	*tar1* - tryptophan aminotransferase related1	Endosperm	(Review) Kai et al. (2021)
			(Review) Dai et al. (2021)
			(Expression) Lu et al. (2019)
Zm00001d037410		Root Elongation Zoom	
Zm00001d041173		Root	
Zm00001d034207		Root	(Expression) Xiang et al. (2022)
Zm00001d033898	*hb36* - Homeobox-transcription factor 36	Leaf	(general) Yilmaz et al. (2009)
Zm00001d018470		Germination Kernal	
Zm00001d041780	*zhd21* - ZF-HD-transcription factor 21	Ear	(transcriptomics) Leiboff et al. (2020)
			(candidate-gene(s)) Liu et al. (2019)
			(general) Yilmaz et al. (2009)
Zm00001d002234	*hb75* - Homeobox-transcription factor 75	Meristem	(gene family) Qiu et al. (2022)
			(promoter) Lee et al. (2021)
			(regulation of expression) Wu et al. (2020)
Zm00001d013130	*bhlh60* - bHLH-transcription factor 60	Leaf	(DEG) Zhao et al. (2021)
			(Expression) Waititu et al. (2021)
			(description) Wu et al. (2019)
Zm00001d042492	*ereb53* - AP2-EREBP-transcription factor 53	Root	(candidate-gene(s)) Ma et al. (2022)
			(gene family) Zhang et al. (2022)
			(Review) Chumakov and Mazilov (2022)

### 3.4 Tissue-specific genes identification by CNN

**FIGURE 5:**
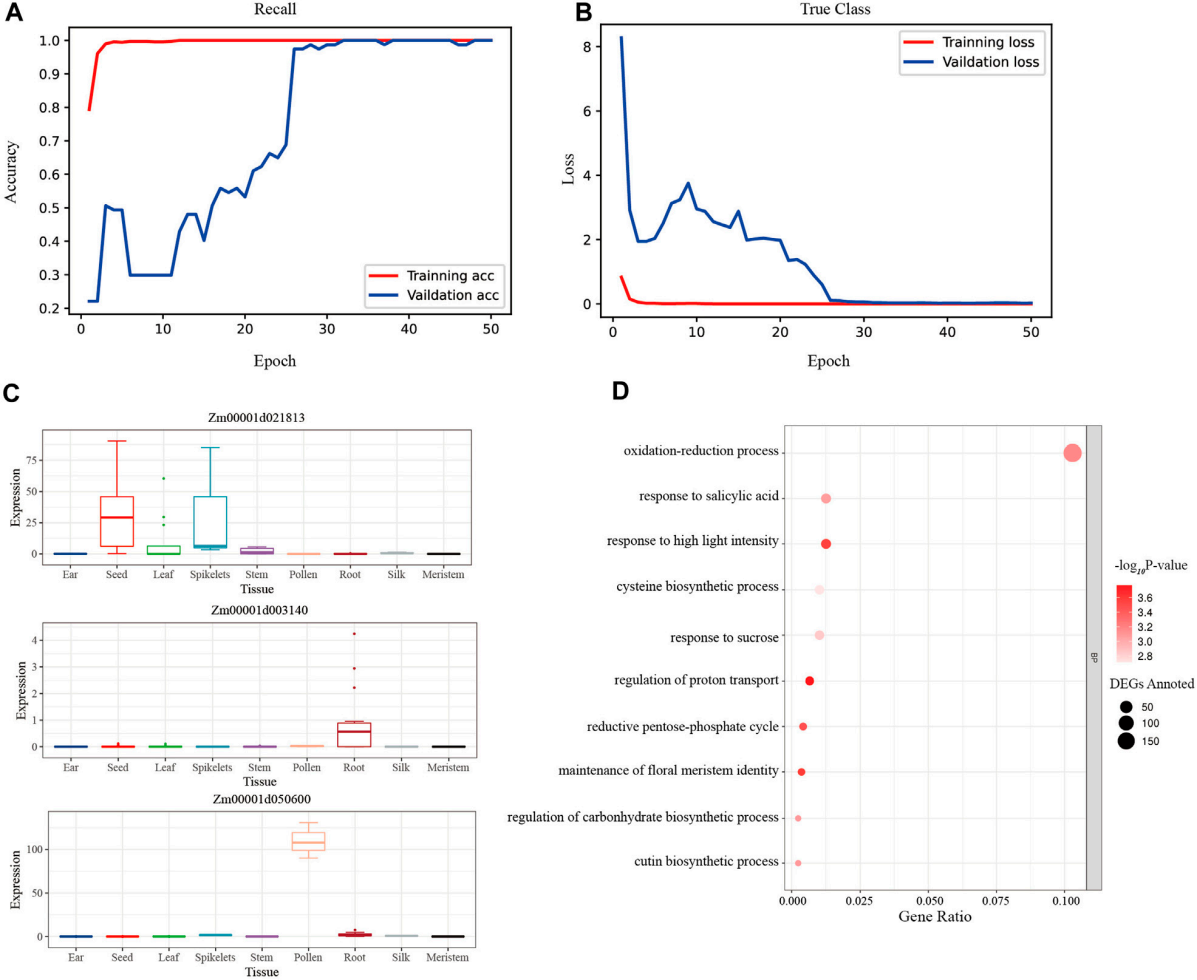
The evaluation plots of CNN model. **(A)** The accuracy rates of the training set and the validation set during the 50 epochs of training. The rate of validation set remained 100% after the 32nd epoch. **(B)** The loss values of the training set and the validation set during the 50 epochs training. The loss values of the validation set remained near 0.25 after the 39th epoch. **(C)** The expression bar plot of the top 3 rank CNN genes queried from the maize expression atlas in MaizeGDB. **(D)** The biological process of the CNN tissue-specific gene set clustered by the GO analysis.

CNN was used to train the modified training set of maize gene expression, and the accuracy rate of the test set remained at 1.00 after the 12th epoch, while the accuracy rate of the validation set remained at 1.00 after the 32nd epoch (Figure 5A). And the loss of the test set reached 3.99 × 10^−6^ in the last epoch, while the loss of the validation set reached 0.0257 finally (Figure 5B). The macro-average F1 score for the test set was 0.91, demonstrating the CNN model’s exceptional robustness and precision. Furthermore, the SHAP values across samples and tissues were calculated, and the high-ranking SHAP genes for each tissue were discovered (Supplementary Table S7).

To assess the biological importance of the high-ranking SHAP genes chosen for each tissue, 1% of the top genes in each tissue were assessed using GO biological processes. The processes of photosynthesis, oxidation-reduction, and epidermal cell differentiation were clustered in the leaf tissue, which represented the vegetative organ (Supplementary Figure S4A); similarly, the processes of stamen development, petal development, and pollen wall assembly were clustered in the tassel tissue, which represented the reproductive organ (Supplementary Figure S4B).

The intersection of the top 50% of unique genes was made using the high-ranking SHAP genes of 11 tissues, yielding a unique collection of 2116 CNN tissue-specific genes. Additionally, an examination of expression bar graphs in MaizeGDB revealed that, as expected, most discovered genes had tissue-specific differential expression (Figure 5C). The filtered genes were clustered in the processes of light stimulus-response, oxidative stress response, pentose-phosphate shunt, and so on using GO analysis (Figure 5D; Supplementary Table S11). This finding revealed that photosynthesis and respiration genes were more likely to be tissue-specific.

### 3.5 Comparison of the three gene set generated from three approaches

**FIGURE 6:**
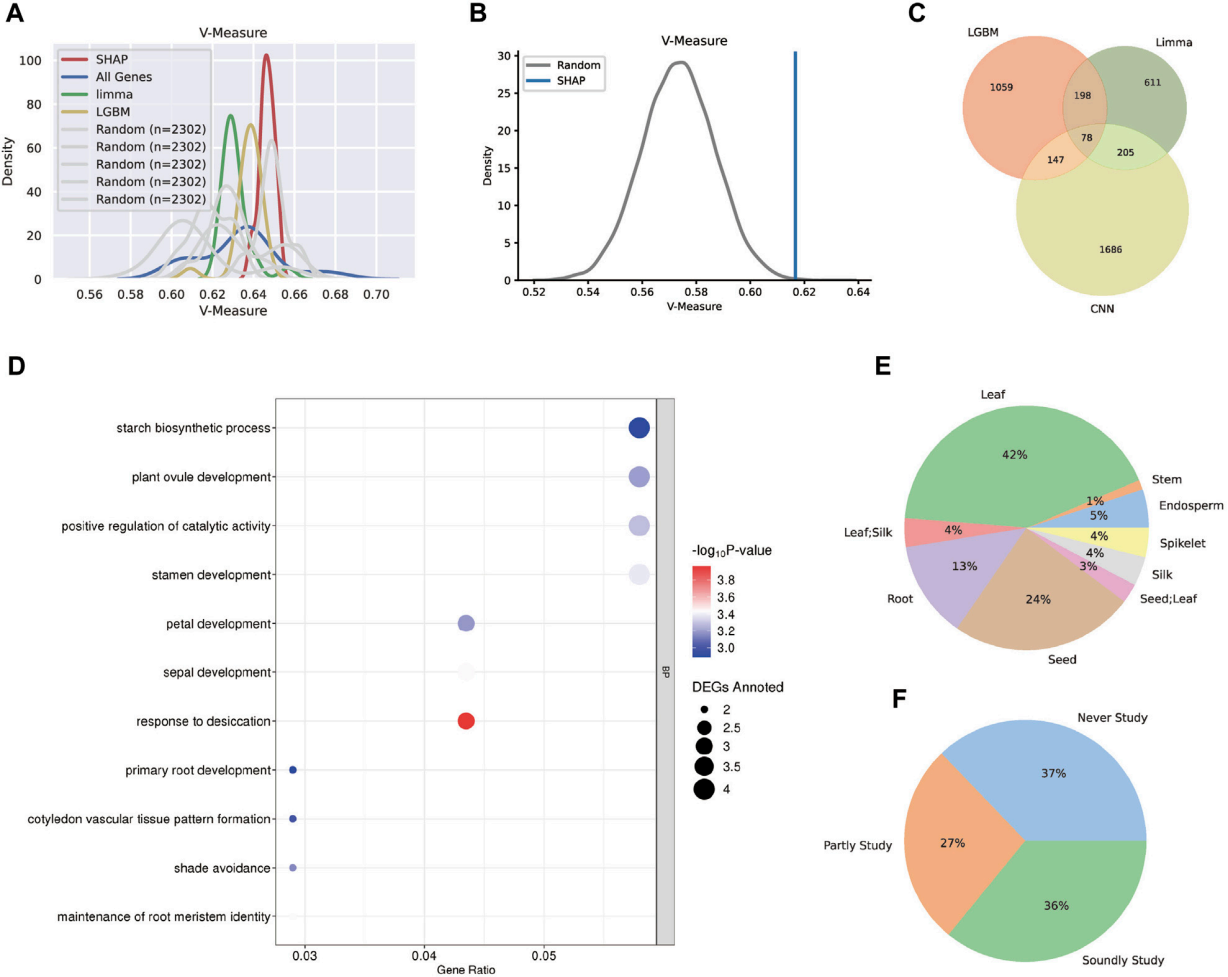
Technical and biological validation for the three gene sets. **(A)** The distribution plot of the V-measure values after k-means clustering. The SHAP tissue-specific gene set performed the best, followed by LGBM and Limma. **(B)** The one-tail Student’s *t*-test estimation between the SHAP gene set and the random gene set. **(C)** The Venn plot for the three tissue-specific gene sets and 78 genes were found in all sets. **(D)** The GO analysis plot for the 78 core genes and most biological processes clustering were tissue-specific. **(E)** The distribution for the tissue types that were specifically expressed by 78 core genes. **(F)** The distribution for the categories reflected the related pieces of literature for 78 core genes. The “soundly study” category had more than three related literature; the “partly study” category had less than three related literature; the “never study” category had no related literature.

The gene sets derived from three unique methodologies were expected to be tissue-specific and capable of differentiating between different tissues’ features. As a result, the k-means approach and the V-measure validation method were used to cluster the samples based on each acquired gene set. SHAP, LGBM, Limma, and total gene V-measure values were 0.647, 0.637, 0.633, and 0.631, respectively, indicating that SHAP genes had a higher V-measure value than other genes (Figure 6A).

Despite the fact that a few randomly chosen gene sets had higher V-measure values, the likelihood of randomly selecting gene subsets that perform as well as the SHAP genes was low, according to one-tail Student’s *t*-test estimation (Figure 6B). Above all, the SHAP gene set was more informative in maize transcriptome data and could distinguish differences between tissues while accurately classifying them.

In the meantime, a number of indices could be used to compare three tissue-specific gene identification methodologies (Table 5), and the outcomes could be analyzed by examining their processing strategies.

(A) Limma was a widely used approach for locating DEGs. This method’s strategy for locating tissue-specific genes was based on the assumption that tissue-specific genes were predominantly DEGs. As a result, tissue-specific genes could be discovered by narrowing the threshold of expression differences, which reflected the biological functions of various tissues. The poor V-measure score of 0.633 can be attributed to the fact that this method could only compare the expression between two sets, which necessitated a lengthy analysis process and failed to account for some non-linear correlations.
(B) LightGBM was able to identify tissue-specific genes using the information entropy gain technique. The gene set based on feature importance scores worked reasonably well in training, resulting in the identification of several essential TFs as high-rank tissue-specific genes. Furthermore, despite having a slightly lower accuracy, the F1 score of LightGBM was higher than CNN, indicating that this tree-based classifier could balance the accuracy and recall rate for classification. However, with an expression matrix of 1,548 rows (samples) and 44,320 columns (genes) as input, the non-neural-network-based machine learning model would easily become overfit.
(C) The CNN model solved the problem of high-dimensionality and overfitting through its structure and was able to precisely predict the tissue type of samples based on their gene expression, with an accuracy of 1. Moreover, its V-measure was predominantly higher than others, indicating that the interpretation of CNN with SHAP could find the most comprehensive tissue-specific gene set, which included the majority of differences between maize tissue expressions and could discriminate samples from different tissue types.

**TABLE 5:** Comparison of three methods in multiple aspects.

Methods	Tissue-specific genes set	Speed	V-measure scores	Prediction accuracy	Prediction F1 score	Interpretation strategy
Limma	1,092	Slow	0.633	None	None	DEGs
LightGBM	1,481	Mediate	0.637	0.9909	0.9908	Information Entropy Gain
CNN	2,116	Fast	0.647	1	0.91	SHAP Values

Aside from the performance of three distinct models, the combined tissue-specific gene sets were also examined, including 78 genes (Figure 6C). According to the GO analysis (Figure 6D), the biological processes of these core set comprehensively show tissue-specific functions, such as starch biosynthetic process for seeds, primary root development and maintenance of root meristem identity for roots, ovule, stamen, petal and sepal development for reproductive organs, and shade avoidance for leaves. Moreover, all core genes were annotated using the maize development atlas (Supplementary Table S12), and their specifically expressing tissues were calculated (Figure 6E), indicating that the majority of the tissue-specific genes were expressed in leaf and seed, with a few of them specifically expressed in two tissues. Furthermore, to assess the dependability of the core set, related literature once investigating these genes was consulted. The genes were classified as “soundly study” or “partly study” based on the number of connected pieces of literature, with genes with no linked literature classified as “never study”. Statistics revealed that 63% of the 78 genes have previously been researched (Figure 6F), indicating that the core gene set in our study was worth examining.

## 4 Discussion

Using high-throughput sequencing techniques, diverse databases of plant transcriptomes comprising integrated sequencing data have been created. The plant databases were created based on a variety of criteria, such as a focus on certain sequencing techniques [PlantExp (Liu et al., 2022), PlantscRNAdb (Chen et al., 2021), etc.] or a focus on individual plant species [Wildsoydb (Xiao et al., 2022), CottonMD (Yang et al., 2022), etc.]. These databases supplied adequate and pertinent data to facilitate machine learning despite information searches and differential analyses between constrained samples. Although researchers have proven that combining RNA-seq with machine learning improves the sensitivity of significant gene discovery, such as DEGs (Ma et al., 2014a), extensive studies to analyze the usefulness and impact of various machine learning approaches in this field have yet to be carried out.

This study incorporated all maize RNA-seq data as an example for comparison analysis with the benchmarking models Limma, LightGBM, and CNN. Among all the prevalent non-neural-network based machine learning models, LightGBM displayed exceptional performance because its AUC values were the highest. Although certain models, such as logistic regression and SVM, had reasonably high accuracy, they may have been hampered by overfitting issues for high-dimensional training matrices and lacked the ability to explain the biological causes behind statistics through interpretation. As a result, LightGBM was chosen to represent the machine learning models, which resulted in a trustworthy tissue-specific gene collection containing several TFs.

Aside from assessing model performance based on technological criteria, it was also important to examine the various tissue-specific gene sets created by different approaches. According to all three gene sets, about 10% of the genes overlapped, which could be explained by the diverse strategies for finding tissue-specific genes. This occurrence was completely consistent with the partially overlapping results of comparison studies on differential expression analysis methods Limma, edgeR, and DESeq2, where Limma utilized a linear model for statistics and the other used the negative binomial distribution (Liu et al., 2021). Concerning Limma, its fundamental premise was based on the detection of DEGs, which were not necessarily tissue-specific and were susceptible to sample size influences. Additionally, because this linear technique could only examine tissue pairs, the comprehensive differences among overall samples were neglected, resulting in lower V-measure scores.

As for LightGBM, it implemented the information entropy theory, which has been demonstrated to be an informative and reliable way for identifying biological genes (Fan et al., 2011; Wallace et al., 2018). The cores of information entropy theory of this model are as follows:

$$\left\{\begin{array}{l} Ent\left(D\right)=-\sum_{k=1}^{\left|y\right|}p_{k}\log_{2}p_{k}\\ Gain\left(D\right)=Ent\left(D\right)-\sum_{v=1}^{V}\frac{\left|D^{v}\right|}{\left|D\right|}Ent\left(D^{v}\right) \end{array}\right.$$ (4),

where p_k_ is the proportion of the samples in different classification, D is the set of the original samples and D^v^ is the set of samples after a branching. |D^v^|/|D| means the weight of the *v*th branch and the Gain(D) indicates the extropy gap before and after splitting. Through this formula, it could be concluded that the feature importance genes, as the classification nodes, are selected during the training period to reach the maximum information gain for every step of classification. The decision tree method would select TFs that played a major role in the gene regulatory network for specific functions as more significant and core nodes to distinguish samples from tissue performing diverse roles. As a result, the LightGBM tissue-specific gene set ranked higher for more well-studied TFs.

Regarding CNN, it avoided the issues of overfitting by rebuilding the expression matrix as input datasets and using SHAP values for interpretation. SHAP values were a post-interpretation method that calculated the marginal contribution of feature genes to model output, which differed from LightGBM’s interpreting-in-progress procedure. SHAP developed an outstanding explanation technique that could not only rank the feature genes based on their contribution scores but also quantify their effects (Supplementary Figure S3); nonetheless, the circumstance where a gene had a high impact on a small number of outputs but a minor impact overall would be overlooked, for only the mediate SHAP value of each gene within each tissue was calculate and ranked. The CNN tissue-specific gene set performed well in the V-measure test overall, but it was less precise and biologically relevant than the LightGBM tissue-specific set. The accuracy of the CNN model would be improved as the increase of the data size and may be superior to traditional machine learning model with outstanding performances in larger datasets (Dhaka et al., 2021). Overall, researchers may use multiple methodologies and strategies for tissue-specific gene sets based on their goals, types of data, and computational resources.

Moreover, the machine learning approach can be applied to linear models as well as classification tasks. Recent advances in machine learning have enabled the development of genotype-phenotypic prediction models that combine transcriptome and phenotype statistics. Cheng et al. used the ensemble learning framework XGBoost to assess the phenotypic diversity of Arabidopsis and maize in terms of nitrogen utilization efficiency and evolutionary conserved transcriptome responses to nitrogen treatment (Cheng et al., 2021). Furthermore, Yan et al. (2021) confirmed LightGBM’s utility in genomic selection-assisted breeding using a large dataset of inbred and hybrid maize lines. LightGBM not only outperformed competitors in prediction accuracy, model stability, and processing efficiency, but it also demonstrated a strong capacity to infer genes that significantly influence phenotypes. In terms of the transcriptome database study, they would be able to predict the phenotype based on expression data and identify significant genes impacting the variance of phenotypes by using numeric features such as the 100 kernels weight and the starch content to train models (Supplementary Figure S5). The list of essential genes might be used with Genome-Wide Association Studies (GWAS) and TFs research to create a data foundation for evaluating crop potential molecular breeding targets.

In conclusion, we demonstrate that the linear Limma method, the machine learning model, and the deep learning model are all valid for identifying tissue-specific genes in maize RNA-seq data. We show that using LightGBM and CNN can find tissue-specific gene sets that perform somewhat better than the linear methodology, as estimated by biologically insight and statistical clustering methods. Our findings show that interpretation models will enable machine learning and deep learning to be applied to large amounts of biological data, potentially yielding new findings. Researchers will be able to fully exploit the plant transcriptome database and get novel insights into plant development and breeding as a result of the ongoing development of high-performance classifiers and dependable methods to explain feature significance.

## Data Availability

The original contributions presented in the study are included in the article/Supplementary Material, further inquiries can be directed to the corresponding author.
