# Supplementary material for: Comparative analysis of tissue-specific genes in maize based on machine learning models: CNN performs technically best, LightGBM performs biologically soundest
Source: Front Genet. 2023 May 9;14:1190887. doi: 10.3389/fgene.2023.1190887 (PMC10203421; doi:10.3389/fgene.2023.1190887)
Supplement: Supplementary file 11 [file DataSheet1.docx]

Supplementary Material

**Comparative analysis of tissue-specific genes in maize based on machine learning models: CNN performs technically best, LightGBM performs biologically soundest**

**Zijie Wang^1*^, Yuzhi Zhu^1^, Zhule Liu^1^**^†^**,** **Hongfu Li^1^**^†^**, Xinqiang Tang^1^**^†^**, Yi Jiang^1^**^†^

^*^Correspondence: Zijie Wang: [wangzj55@mail2.sysu.edu.cn](mailto:wangzj55@mail2.sysu.edu.cn)

^†^These authors contributed equally to this work and share last authorship.

1. **Supplementary Tables**

**S1 Table. The sample information of the maize RNA-seq was obtained from NCBI and ENA.**

**S2 Table. The simplified maize expression matrix.**

https://doi.org/10.6084/m9.figshare.22309180

**S3 Table. The DEGs found by Limma.** Different sheets of the table referred to different tissue pairs.

**S4 Table. The Limma tissue-specific gene set.**

**S5 Table. The list of important genes found by LightGBM during the training process.**

**S6 Table. The LGBM tissue-specific gene set.**

**S7 Table. The SHAP values between all samples and tissues.**

https://doi.org/10.6084/m9.figshare.22309189

**S8 Table. The SHAP tissue-specific gene set.**

**S9 Table. The GO analysis result of Limma tissue-specific genes.**

**S10 Table. The GO analysis result of LGBM tissue-specific genes.**

**S11 Table. The GO analysis result of CNN tissue-specific genes.**

**S12 Table. The annotation for 78 core tissue-specific genes**

1. **Supplementary Figures**

**
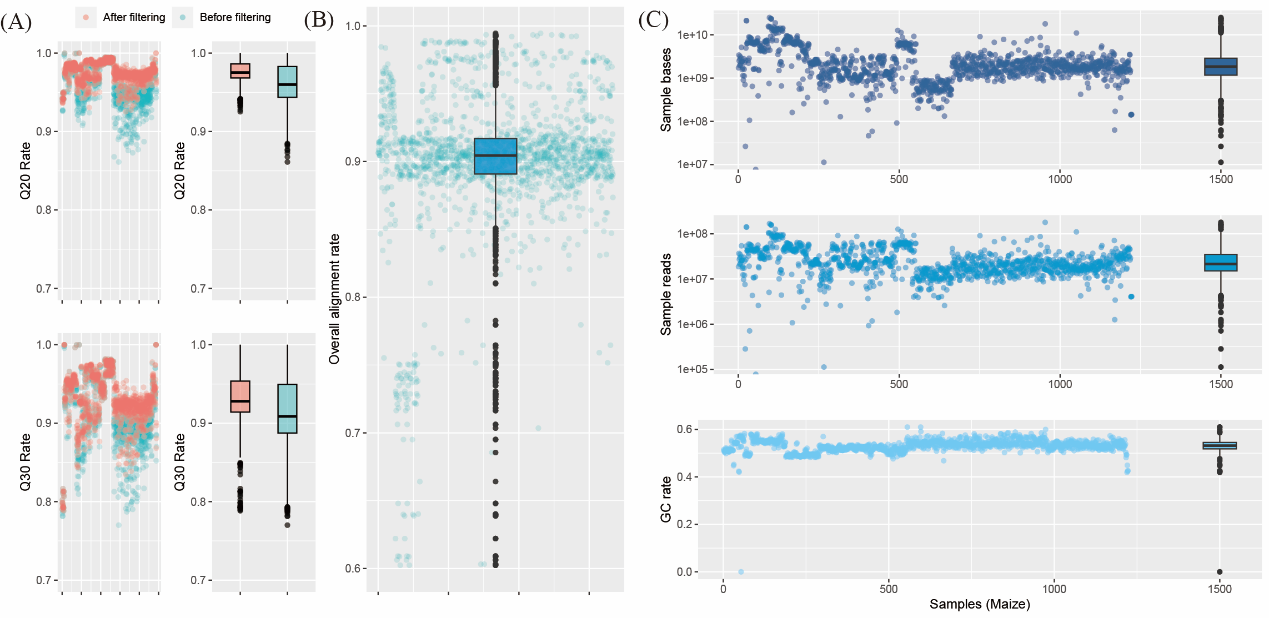
**

**S1 Fig. The results of maize RNA-seq data processing.** (A) The Q20 and Q30 filtered rate of Fastp processing. (B) The overall alignment rate for 1548 maize RNA-seq. (C) The sample bases, sample reads and GC rate for 1548 maize RNA-seq.


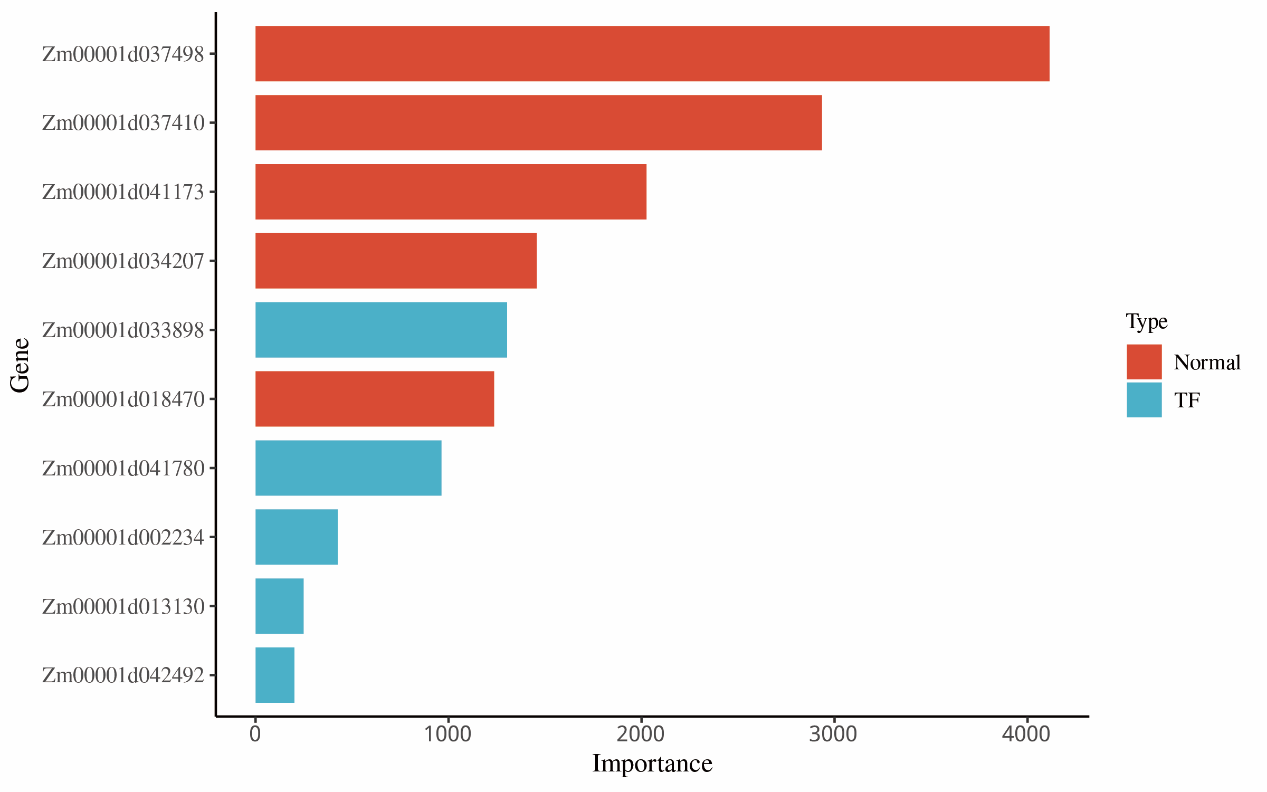


**S2 Fig. The top 10 feature importance genes in LightGBM model.**

**
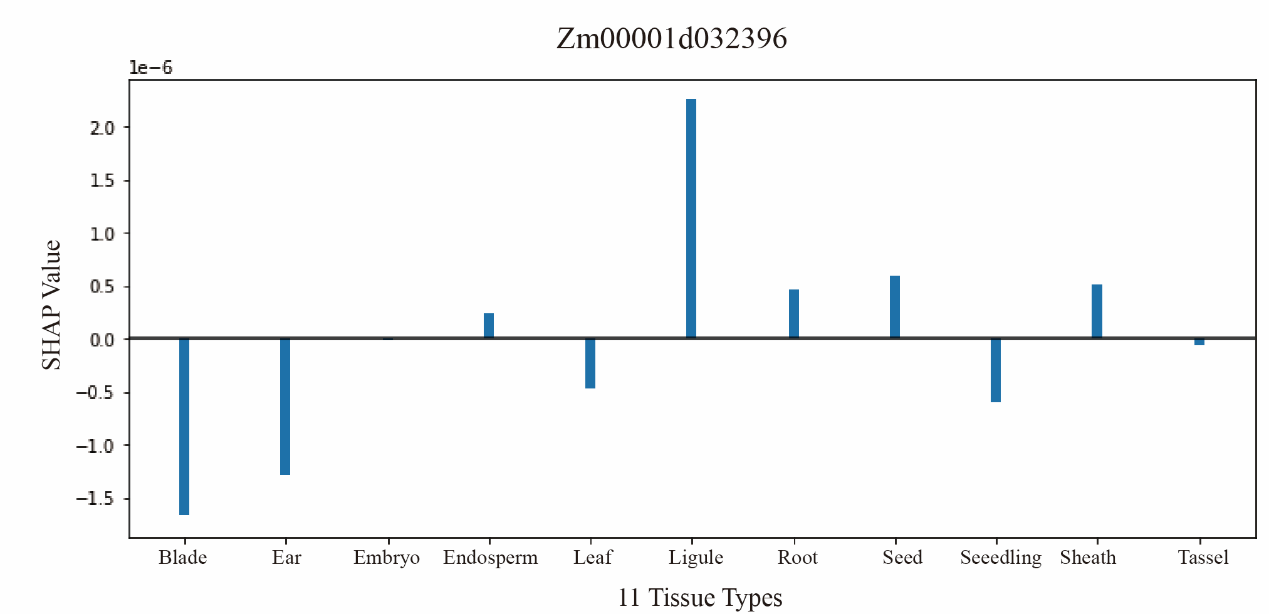
**

**S3 Fig. The SHAP values of Zm00001d032396 across 11 tissue types.** The SHAP values could reflect the positive or negative contributions of the gene to the tissue types prediction.

**
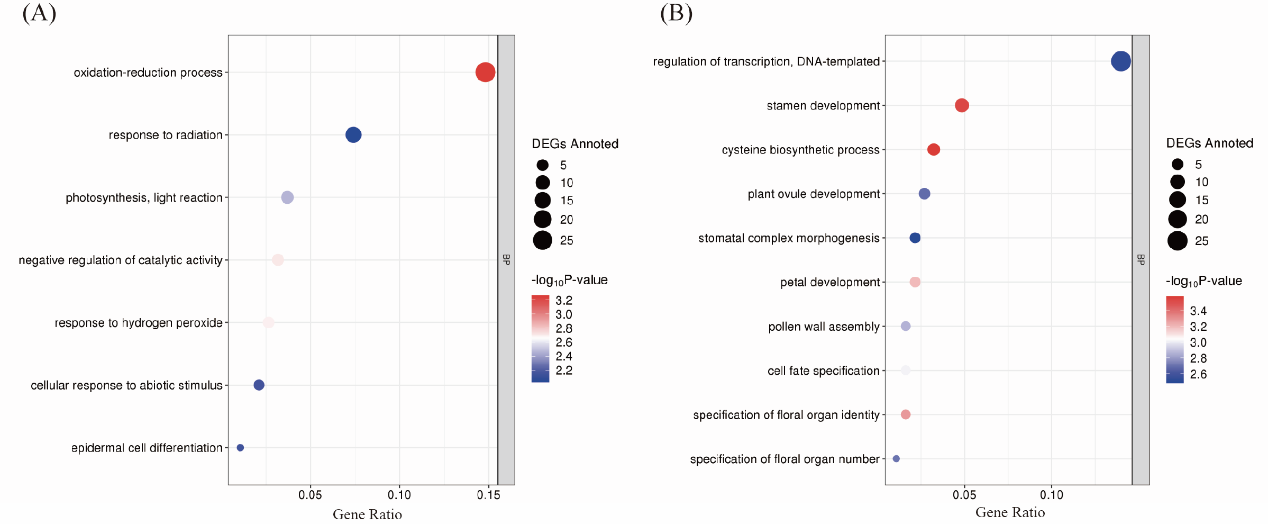
**

**S4 Fig. Biological analysis of the high-ranking SHAP genes and the CNN gene set.** (A) The GO analysis result of the high-rank SHAP genes in leaf tissue. (B) The GO analysis result of the high-rank SHAP genes in tassel tissue.

**
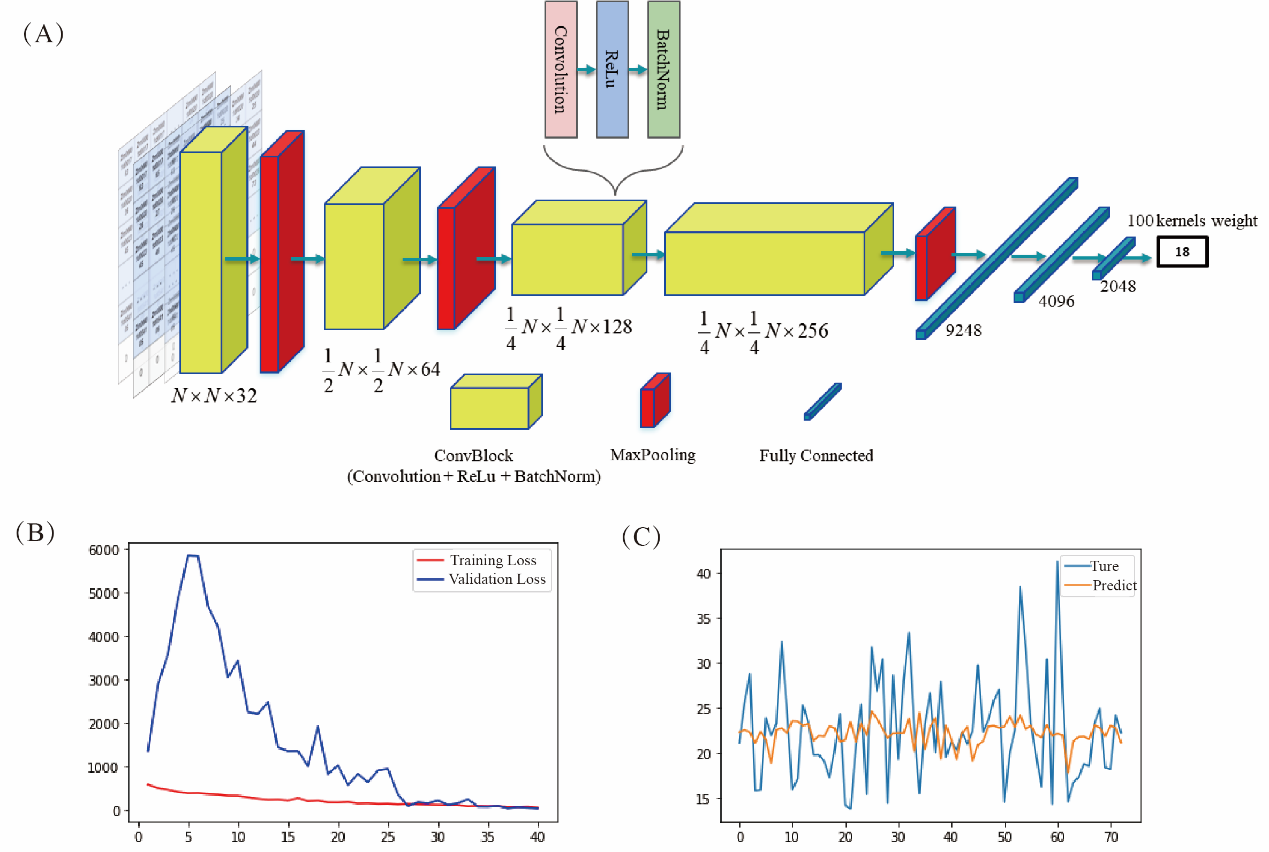
**

**S5 Fig. The CNN model for predicting the 100 kernels weight of maize according to the expression data.** (A) The architecture of the CNN model. The output layers were modified from the classification model. (B) The loss curve of training set and validation set during the 40 epochs training. (C) The distributions of prediction values and the test values in the test set.

1. **Code availability**

The codes of CNN training, SHAP interpretive model and V-measure validation are available at **https://github.com/Wong718/CNN-for-transcriptome**.
